# Telemonitoring to Manage Chronic Obstructive Pulmonary Disease: Systematic Literature Review

**DOI:** 10.2196/11496

**Published:** 2019-03-20

**Authors:** Clemens Kruse, Brandon Pesek, Megan Anderson, Kacey Brennan, Hilary Comfort

**Affiliations:** 1 School of Health Administration Texas State University San Marcos, TX United States

**Keywords:** telemedicine, COPD, chronic disease

## Abstract

**Background:**

Chronic obstructive pulmonary disease (COPD) is a leading cause of death throughout the world. Telemedicine has been utilized for many diseases and its prevalence is increasing in the United States. Telemonitoring of patients with COPD has the potential to help patients manage disease and predict exacerbations.

**Objective:**

The objective of this review is to evaluate the effectiveness of telemonitoring to manage COPD. Researchers want to determine how telemonitoring has been used to observe COPD and we are hoping this will lead to more research in telemonitoring of this disease.

**Methods:**

This review was conducted in accordance with the Assessment for Multiple Systematic Reviews (AMSTAR) and reported in accordance with the Preferred Reporting Items for Systematic Reviews and Meta-Analyses (PRISMA). Authors performed a systematic review of the PubMed and Cumulative Index to Nursing and Allied Health Literature (CINAHL) databases to obtain relevant articles. Articles were then accepted or rejected by group consensus. Each article was read and authors identified barriers and facilitators to effectiveness of telemonitoring of COPD.

**Results:**

Results indicate that conflicting information exists for the effectiveness of telemonitoring of patients with COPD. Primarily, 13 out of 29 (45%) articles stated that patient outcomes were improved overall with telemonitoring, while 11 of 29 (38%) indicated no improvement. Authors identified the following facilitators: reduced need for in-person visits, better disease management, and bolstered patient-provider relationship. Important barriers included low-quality data, increased workload for providers, and cost.

**Conclusions:**

The high variability between the articles and the ways they provided telemonitoring services created conflicting results from the literature review. Future research should emphasize standardization of telemonitoring services and predictability of exacerbations.

## Introduction

### Rationale

The most recent estimate of the world prevalence for chronic obstructive pulmonary disease (COPD) is 64 million, with 3 million deaths from the disease in 2015 alone [1]. The World Health Organization (WHO) estimates that COPD will be the third-leading cause of death in the world by 2030 and that 90% of its victims live in middle-to-low-income countries [1]. It is primarily caused by cigarette smoke—primary or secondary—and exacerbated by long-term asthma [1]. The United States addressed the increase in prevalence by penalizing reimbursement for public health beneficiaries if a hospital readmitted the patient for the condition within 30 days [2].

The United States also passed the Health Information Technology for Economic and Clinical Health (HITECH) Act in 2009, which incentivized the adoption of health information technology up until 2015 and penalized the lack of adoption thereafter [3]. The HITECH Act served as a catalyst for the diffusion of telemedicine in the United States, which is important because the United States lagged behind other western nations in the use and acceptance of telemedicine. There are many facets to telemedicine, but we will start with a general definition.

The WHO defines telemedicine as follows [4]:

The delivery of health care services, where distance is a critical factor, by all health care professionals using information and communication technologies for the exchange of valid information for diagnosis, treatment and prevention of disease and injuries, research and evaluation, and for the continuing education of health care providers, all in the interests of advancing the health of individuals and their communities.

We choose this definition for our review and, also following the WHO’s example, we do not distinguish between telemedicine and telehealth.

US national attention on telemedicine services for rural and other low-access populations has steadily increased in the past decade. As the United States continues to determine how best to fund these services and how to legislate accreditation across state borders and specialties, much research is being conducted on the efficacy of various telemedicine services [5]. While the bulk of US research attends to clinical interventions provided for mental health and chronic diseases, chronic diseases also require regular monitoring of health parameters.

Telemedicine, in its modern form, developed through rapid advancement in communication technology and innovation on the part of health care professionals [6]. Naturally, physicians treating chronic diseases, such as COPD, required methods to track patient health factors and telemonitoring was the solution. Telemonitoring is defined as the distance monitoring of components of a patient’s health as part of a larger chronic care model [7]. These methods, when applied to patients with COPD, can utilize caregiver review of data to assess disease state and health status [8]. Telemonitoring of COPD even has the potential to predict exacerbations before onset [9].

### Objective

The objective of this review is to evaluate the effectiveness of telemonitoring to manage the chronic disease of COPD. We want to look at how telemonitoring has been used to observe COPD and we are hoping this will lead to more research in telemonitoring of this disease. We used techniques from the Assessment for Multiple Systematic Reviews (AMSTAR) and reported our findings in accordance with the Preferred Reporting Items for Systematic Reviews and Meta-Analyses (PRISMA) [10,11].

## Methods

### Stakeholder Involvement in the Review

Neither patients, service users, caregivers, nor lay people were used in the design or execution of this review. The development of outcome measures was not informed by patients’ priorities, experience, or preferences. Neither patients, caregivers, nor lay people were involved in the recruitment to and conduct of this review. Since there were no study participants as part of this review, dissemination of results to participants was unnecessary. The development of the research question and outcome measure was not informed by patients’ priorities, experience, or preferences. Because there were no study participants, ethics approval and consent to participate were not necessary.

### Protocol, Registration, and Information Sources

This systematic literature review followed standard retrieval methods to obtain peer-reviewed articles: multiple-reviewer technique set by the AMSTAR standard to evaluate them and the PRISMA standard to report the analysis conducted on the articles in the review [12,13]. Authors queried the following databases: PubMed and the Cumulative Index to Nursing and Allied Health Literature (CINAHL), which is managed by EBSCO. Authors used the search terms *telemonitoring* and *COPD* and all associated Medical Subject Headings (MeSH) terms: *Chronic Obstructive Pulmonary Disease, COAD, Chronic Obstructive Airway Disease, Chronic Obstructive Lung Disease, Airflow Obstruction, Chronic or Airflow Obstructions, Chronic or Chronic Airflow Obstruction,* and *Chronic Airflow Obstruction*. This review was not registered.

### Study Selection and Data Collection Process

Databases were searched for articles published during the time frame of February 1, 2011, through February 1, 2017. Originally, we planned to limit our search to a 5-year span for analysis because of the rapid advancement of technology, but this did not yield a suitable number of articles to analyze. As a result, we expanded our search to a 6-year span. Boolean operators were used during searches to obtain the desired search parameters.

The initial search in PubMed, *telemonitoring* AND *COPD*, returned 88 articles. Restricting the articles further by date eliminated 12 articles; limiting by academic journals and English-only articles eliminated 21 more articles. The initial search in CINAHL generated a total of 38 articles. Restricting the publication date range minimized this number to a total of 35 articles and removing any articles that were not from academic journals and not written in English resulted in 16 remaining articles.

### Data Items and Eligibility Criteria

Authors created a literature matrix detailing the title, author, year, journal, and other pertinent information of the 61 articles in preparation for final screening. To eliminate the possibility for bias, two authors read each abstract and came to a consensus regarding whether the article was germane to the topic. Reviewers agreed that for an article to be accepted for analysis, it had to be published in the last 6 years in a peer-reviewed journal and it had to provide substantive data on the use of telemonitoring to manage COPD. Once all abstracts had been screened for suitability, the authors used a consensus meeting to make final determination on whether to eliminate articles that were not germane to the specific topic and remove any duplicate articles. Through this process, the authors noticed a common reference that had not been caught in their query of the databases. The final number of articles gathered from PubMed and CINAHL totaled 21 and 8, respectively, bringing the final combined total to 29 articles for analysis in the literature review. A kappa statistic was calculated to determine interrater reliability (.91), which is indicative of strong agreement [14,15].

Authors read each article closely and made independent notes of common themes related to each review’s objective. These were shared at a second consensus meeting; through a brief discussion of content and findings, detailed notes were highlighted about barriers and facilitators to the adoption of telemonitoring for the management of COPD. Frequency of occurrence of each of the barriers and facilitators were captured in affinity matrices for further analysis. Data and calculations are available upon request.

## Results

### Study Selection and Study Characteristics

**Figure 1:**
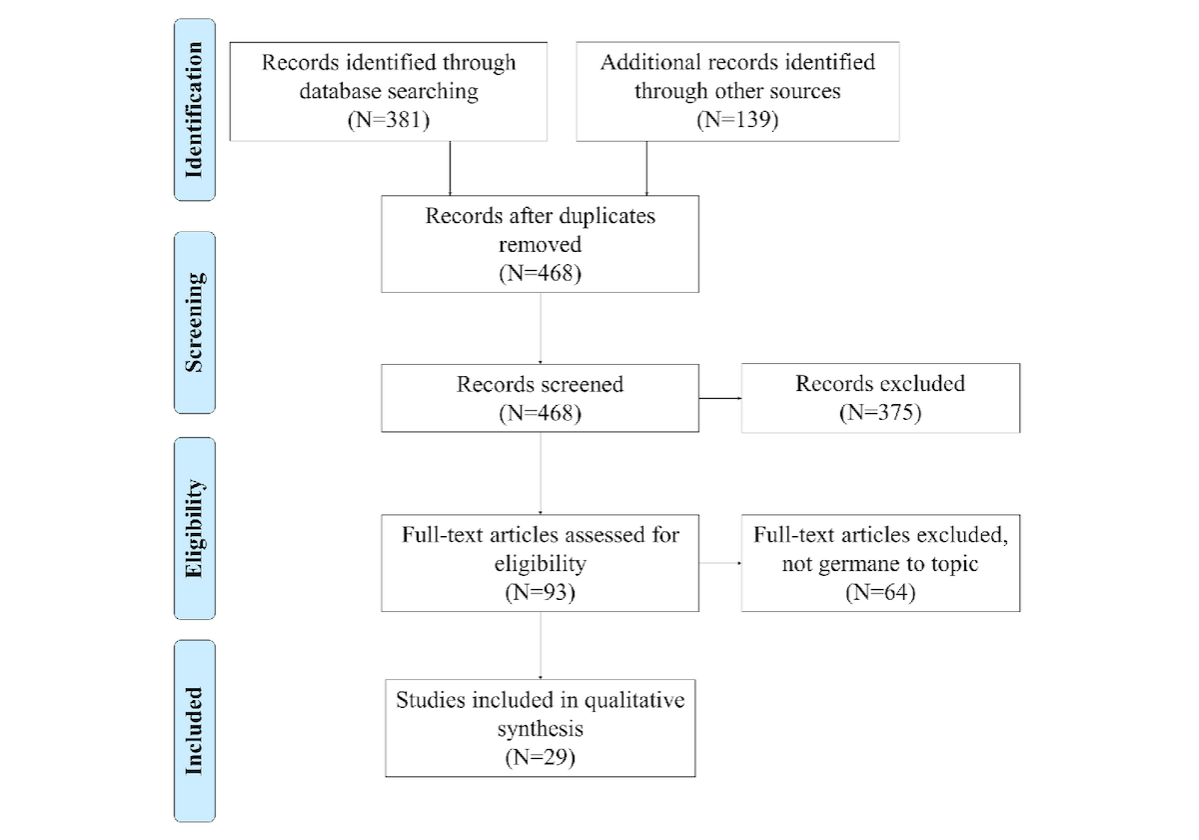
Literature search and selection process.

From the original 136 articles resulting from the initial search, 97 were screened out due to date of publication, nature of publication, and whether the topic was germane to our research (ie, possibly indexed improperly). The list of germane studies was narrowed down to 29. The literature search process is listed in Figure 1.

### Results of Individual Studies

The results were mixed regarding the efficacy of telemonitoring to reduce complications associated with COPD. Any clear positive relationship with the use of telemonitoring to manage COPD was obscured. A list of all 29 studies [8,9,16-42] and a summary of each topic is listed in Table 1.

**Table 1:** Summary of articles analyzed and applicable observations.

Authors and reference	Summary of topic
Jensen MH et al [8]	Clinical impact of home telemonitoring on patients with COPD^a^
Sanchez-Morillo D et al [9]	Pilot study detecting COPD exacerbations early using daily telemonitoring of symptoms and k-means clustering
Alrajab S et al [16]	A home telemonitoring program reduced exacerbation and health care utilization rates in COPD patients with frequent worsening of symptoms
Antoniades NC et al [17]	Pilot study of remote telemonitoring in COPD
Burton C et al [18]	Changes in telemonitored physiological variables and symptoms prior to exacerbations of chronic obstructive pulmonary disease
Celler BG and Sparks RS [19]	Home telemonitoring of vital signs—technical challenges and future directions
Chatwin M et al [20]	Randomized crossover trial of telemonitoring in chronic respiratory patients (TeleCRAFT trial)
Davis C et al [21]	Feasibility and acute care utilization outcomes of a post-acute transitional telemonitoring program for underserved chronic disease patients
Elwyn G et al [22]	Detecting deterioration in patients with chronic disease using telemonitoring: navigating the “trough of disillusionment”
Fairbrother P et al [23]	Exploring telemonitoring and self-management by patients with chronic obstructive pulmonary disease: a qualitative study embedded in a randomized controlled trial
Fairbrother P et al [34]	Continuity, but at what cost? The impact of telemonitoring COPD on continuities of care: a qualitative study
Fernandez-Granero MA et al [25]	Automatic prediction of chronic obstructive pulmonary disease exacerbations through home telemonitoring of symptoms
Fernandez-Granero MA et al [26]	Computerized analysis of telemonitored respiratory sounds for predicting acute exacerbations of COPD
Goldstein RS and O'Hoski S [27]	Telemedicine in COPD: time to pause
Ho TW et al [28]	Effectiveness of telemonitoring in patients with chronic obstructive pulmonary disease in Taiwan: a randomized controlled trial
Jordan R et al [29]	Telemonitoring for patients with COPD
Kim J et al [30]	Acceptability of the consumer-centric uHealth services for patients with chronic obstructive pulmonary disease
Kim J et al [31]	Effects of consumer-centered uHealth service for the knowledge, skill, and attitude of the patients with chronic obstructive pulmonary disease
Martin-Lesende I et al [32]	Impact of telemonitoring home care patients with heart failure or chronic lung disease from primary care on health care resource use (ie, the TELBIL study randomized controlled trial)
McDowell JE et al [33]	A randomized clinical trial of the effectiveness of home-based health care with telemonitoring in patients with COPD
McKinstry B [34]	The use of remote monitoring technologies in managing chronic obstructive pulmonary disease
Pedone C et al [35]	Efficacy of multiparametric telemonitoring on respiratory outcomes in elderly people with COPD: a randomized controlled trial
Pedone C and Lelli D [36]	Systematic review of telemonitoring in COPD: an update
Pinnock H et al [37]	Effectiveness of telemonitoring integrated into existing clinical services on hospital admission for exacerbation of chronic obstructive pulmonary disease: researcher-blind, multicenter, controlled trial
Reddel HK et al [38]	Self-management support and other alternatives to reduce the burden of asthma and chronic obstructive pulmonary disease
Stoddart A et al [39]	Telemonitoring for chronic obstructive pulmonary disease: a cost and cost-utility analysis of a randomized controlled trial
Venter A et al [40]	Results of a telehealth-enabled chronic care management service to support people with long-term conditions at home
Vianello A et al [41]	Home telemonitoring for patients with acute exacerbation of chronic obstructive pulmonary disease: a randomized controlled trial
Zanaboni P et al [42]	Long-term telerehabilitation of COPD patients in their homes: interim results from a pilot study in Northern Norway

^a^COPD: chronic obstructive pulmonary disease.

**Table 2:** Facilitators to the adoption of telemedicine to manage chronic obstructive pulmonary disease (COPD).

Facilitators	Articles	Occurrence (N=56), n (%)
Improved patient outcomes or satisfaction	[8,9,16,17,24,29-31,36,38,40]	13 (23)
Reduced need for in-person visits	[8,9,16,17,22,30,32,35,36]	9 (16)
Better disease management	[8,17,22,28,30,31,34,40]	8 (14)
Bolstered patient-provider relationship	[20,21,23,24,30]	5 (9)
High-quality data	[16,19,25,28]	4 (7)
Patient empowerment	[8,17,21,23]	4 (7)
Ease of use	[17,25,32]	3 (5)
Predictability of exacerbations	[9,19,25]	3 (5)
Provision of additional services	[30,32,41]	3 (5)
Patient engagement	[16,17]	2 (4)
Access to patient data	[16]	1 (2)
Communication	[22]	1 (2)

**Table 3:** Barriers to the adoption of telemedicine to manage chronic obstructive pulmonary disease (COPD).

Barriers	Articles	Occurrence (N=57), n (%)
Reduced patient outcomes or no improvement	[16-18,20,28,31,38-42]	11 (19)
Low-quality or limited data	[8,16-18,22-25,28]	9 (16)
Increased workload for providers	[20,19,24,30,32,35,37]	7 (12)
Cost	[29-31,34,39]	5 (9)
Heterogeneity of care	[6,8,21,34,42]	5 (9)
Lack of service standardization	[18,21,22,24,28]	5 (9)
Exacerbations are highly variable	[9,16,18,34]	4 (7)
Uncomfortable with technology	[22,31,33]	3 (5)
Less patient autonomy	[9,20,21]	3 (5)
Time-consuming	[22,24]	2 (4)
Staff shortages or overworked staff	[8]	1 (2)
User perception or perceived lack of usefulness	[23]	1 (2)
User or patient resistance	[24]	1 (2)

Our second consensus meeting helped us identify the 12 facilitators and 13 barriers to the acceptance and feasibility of telemonitoring to manage COPD, which are summarized in Tables 2 and 3, respectively.

Facilitators and barriers are sorted by frequency of occurrence. We do not suggest that frequency equates to importance; we highlight only the probability that each theme occurred in the literature.

### Synthesis of Results and Risk of Bias Across Studies

Throughout this review, the value of telemonitoring to manage COPD symptoms has been intensively evaluated. Authors identified key facilitators and barriers related to the effectiveness of telemonitoring. The prevalence of factors can be reviewed in Tables 2 and 3. Conflicting data were found detailing the efficacy of telemonitoring services for managing COPD. Some articles cited improvements in patient outcomes, satisfaction, anxiety and depression, and hospitalization rates in the facilitator *improved patient outcomes or satisfaction* [8,9,16,17,24,29-31,33,34,36,38,40], while others stated that no significant improvement occurred under the barrier *reduced patient outcomes or no improvement* [16-18,20,28,31,38-42].

Articles discuss various causes for improvement of the COPD disease state or perceptions of the disease state. A total of 31% (9/29) of articles stated that telemonitoring reduced the number of in-patient visits required for patients engaged in telemonitoring care, including primary care visits and emergency department visits [8,9,16,17,22,30,32,35,36]. Pinnock [37] and Venter [40] found that enhanced access to care was especially useful in rural areas where access to care may be greatly restricted. As a dominant facilitator for telemonitoring of COPD, the review suggests that telemonitoring interventions have the potential to achieve the main goal of telemedicine services.

A common reason for patient improvements included that providing telemonitoring services to traditional COPD management added underlying services lines to patient resources [32,34,41]. As more service options were added, including videoconferencing and phone support, articles noted reductions in admissions related to exacerbations. McKinstry [34] found that more successful programs were associated with service lines that were unavailable to regular COPD management programs. Constant access to a respiratory nurse should logically increase patient education and outcomes. Regardless of the number of added service lines provided through telemonitoring of COPD, patients were regularly satisfied with the telemonitoring services provided. Other facilitators, such as higher-quality patient data and ease of use, provide better self-management for patients and more information to caregivers.

Conversely, numerous articles in this review also mentioned the inability for COPD telemonitoring to provide added value for patients [16-18,20,28,31,38-42]. Some of these articles referenced that sample selection did not allow for clear improvements; authors, in some instances, selected patients with excellent self-management practices [36]. Authors also reported that as telemonitoring services expanded, clinician and nurse workloads increased [20,23,24,32,34,37,39]. As staffing is one of the most expensive parts of providing health care, increasing the amount of work required to care for patients can potentially increase costs.

Cost also factored into some of the studies examined [33-35,38,39]. Results ranged from incremental cost-effectiveness ratios of £203,900 to descriptions of increased cost of care [33]. With 17% (5/29) of studies referencing no improvement or reduced patient outcomes, the literature suggests that caregivers hesitate before providing telemonitoring care that is not cost-effective. Other barriers to consider are usability of devices, perceived lower autonomy of patients, and time required to obtain symptom data.

A total of 3 articles out of 29 (10%) explained prediction methods to determine the onset of exacerbations [9,19,25]. One article by Sanchez-Morillo et al [9] predicted 93.9% of exacerbations within 4.5 days of necessary medical interventions; they showed that improved patient outcomes and reduced in-patient visits can be achieved even with high variability of exacerbations. This article illustrates the variety of telemonitoring interventions available and an ideal method of protecting patient health through telehealth services.

## Discussion

### Principal Findings

A total of 12 facilitators [8,9,16,17,22,24-32,38,40] and 13 barriers [8,9,16-25,28,31-42] were identified in the literature and a total of 113 occurrences were detected. While frequency does not impute importance, it does identify those issues most salient to those authors during the 6 years within which studies were published. Multiple factors were identified as both facilitators and barriers, but not by the same authors. The results of this review do not conflict with findings of other reviews, but they do imply several issues for consideration in health policy. Cost continues to play a reduced role in the use of telemedicine, which is a positive trend [33-35,38,39]. Policy makers should continue current incentives, but realize this may affect fewer organizations as the cost of implementation has already been absorbed. Lack of standardization is a barrier of concern and this issue is being addressed through organizations making and developing standards [18,21,22,24,28]. The most concerning and most frequently mentioned barrier was reduced patient outcomes or no improvement [16-18,20,28,31,38-42]. Technology is already expensive and it is often more complex than traditional care; decision makers seldom choose to pursue an intervention with technology unless there are improved patient outcomes that offset the cost of the technology itself. Policy makers need to carefully endorse those technology-infused interventions that yield positive patient outcomes and recommend that developers work on the rest of the barriers until the threshold for improvement is crossed.

### Limitations

Authors noted some minor limitations. The high variability between articles, patient samples, telemonitoring methods, and treatments may explain why 24% (7/29) of the articles found improved patient outcomes and 21% (6/29) found no improvement in outcomes. With such differences between studied effects, external validity of the literature may be compromised. Further, because researchers were responsible for determining which articles were included in the study, readers should be aware of selection bias. However, biases were controlled by utilizing multiple reviewers for each article who discussed inclusion or exclusion of articles, as discussed in the Methods section.

### Conclusions

Authors determined that many conflicting barriers and facilitators exist to the adoption of telemonitoring for patients with COPD. Due to the high variability of patients monitored, service lines, types of technology, and severity of disease state, some studies do not relate well to others. Future research should emphasize the importance of standardizing the telemonitoring of COPD techniques and the ability of technology to predict exacerbations. Predictability of exacerbations, even with the large range of pre-exacerbation symptoms, will reduce in-person visits and provide patients with useful warnings about their conditions. Through continued evaluation of COPD efficacy, research may find a cost-effective and useful standard for monitoring COPD through telehealth interventions.

## Abbreviations

AMSTAR: Assessment for Multiple Systematic Reviews
CINAHL: Cumulative Index to Nursing and Allied Health Literature
COPD: chronic obstructive pulmonary disease
HITECH: Health Information Technology for Economic and Clinical Health
MeSH: Medical Subject Headings
PRISMA: Preferred Reporting Items for Systematic Reviews and Meta-Analyses
WHO: World Health Organization
